# An integrated protein structure fitness scoring approach for identifying native-like model structures

**DOI:** 10.1016/j.csbj.2022.11.032

**Published:** 2022-11-17

**Authors:** Rahul Kaushik, Kam Y.J. Zhang

**Affiliations:** Laboratory for Structural Bioinformatics, Center for Biosystems Dynamics Research, RIKEN, 1-7-22 Suehiro, Yokohama, Kanagawa 230-0045, Japan

**Keywords:** Protein structure scoring function, Structure quality assessment, Protein structure modelling, Machine learning, Computational protein folding, Computational protein design

## Abstract

The structural information of a protein is pivotal to comprehend its functions, protein–protein and protein–ligand interactions. There is a widening gap between the number of known protein sequences and that of experimentally determined structures. The protein structure prediction has emerged as an efficient alternative to deliver the reliable structural information of proteins. However, it remains a challenge to identify the best model among the many predicted by one or a few structure prediction methods. Here we report ProFitFun-Meta, a neural network based pure single model scoring method for assessing the quality of predicted model structures by an effective combination structural information of various backbone dihedral angle and residue surface accessibility preferences of amino acid residues with other spatial properties of protein structures. The performance of ProFitFun-Meta was validated and benchmarked against current state-of-the-art methods on the extensive datasets, comprising a Test Dataset (n = 26,604), an External Dataset (n = 40,000), and CASP14 Dataset (n = 1200). The comprehensive performance evaluation of ProFitFun-Meta demonstrated its reliability and efficiency in terms of Spearman’s (ρ) and Pearson’s (r) correlation coefficients, GDT-TS loss (g), and absolute loss (d). An improved performance over the current state-of-the-art methods and leading performers of CASP14 experiment in quality assessment category demonstrated its potential to become an integral component of computational pipelines for protein modeling and design. The minimal dependencies, high computational efficiency, and portability to various Linux and Windows OS provide an additional edge to ProFitFun-Meta for its easy implementation and applications in various regimes of computational protein folding.

## Graphical abstract

**Figure:**
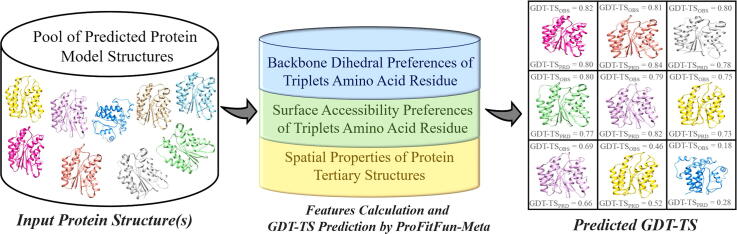

## 1 Introduction

The recent development in high-throughput next-generation sequencing techniques and in contrast to the time-consuming experimental methods for protein structure determination has widened the gap between experimentally solved protein structures and known protein sequences [1], [2], [3], [4]. The structural information of a protein is pivotal to comprehend its functions, protein–protein and protein–ligand interactions and several other properties. Subsequently, the protein structure prediction has emerged as an efficient alternative to deliver the reliable structural information of proteins to substantiate various aspects of computational protein design, computer aided drug discovery, and protein functional annotations [5], [6], [7]. The latest development of advanced deep-learning based methods has revolutionized the field of protein structural modeling, as showcased through CASP (Critical Assessment of Structure Prediction) and CAMEO (Continuous Automated Model Evaluation) experiments [3], [4], [8], [9], [10]. These new methods have been used to efficiently complement the experimental methods to narrow the gap between available protein structural information and sequences [6], [11], [12], [13]. The prediction accuracies of the protein structural modeling methods, implementing different algorithms for predicting the 3D atomic model structures from the amino acid sequences, varies case by case. Consequently, the end user is often perplexed by having topologically diverse multiple models from different state-of-the-art-methods for the same protein sequence. This problem of having many candidate structures further intensifies if there is hardly any structure related information at disposal for a target protein sequence [7], [14], [15], [16]. Almost all the protein structure prediction methods generate numerous model structures (also called decoys) for the target protein sequence, by implementing different approaches, such as *de novo* methods, homology-based methods, hybrid methods. Depending on the approach used and availability of computational resources, the number of decoys may vary from hundreds to thousands [5], [9], [11], [17]. These decoys are further screened to identify the best model structure(s) that could be deputized as the most suitable structural substitute for the protein sequence. Hence, an exceedingly efficacious measure to scrutinize the decoys is an obligatory ingredient to consummate a desirable protein structure prediction.

Primarily, the methods for protein structure quality assessment employ-three approaches, either individually or in combination, viz. pure single model, quasi single model and clustering based assessment approach [18], [19], [20], [21], [22], [23]. In the pure single model approach, the individual decoy is evaluated regardless of all other decoys or availability of any experimentally solved homologous protein structure. While in quasi single model approach, the best decoy is singled out from all the decoys by harnessing the pure single model approach and its structural details are utilized to score and rank order the other decoys. In the clustering approach, a consensus criterion is deduced from the similarity among the decoys and exercised to score all the decoys.

Over the years, the scientific community has witnessed the development of several state-of-the-art methods for assessing the decoys to distinguish the most suitable model structures with a reasonable accuracy. The assessment from recent CASP experiments revealed the superior accuracy of consensus approaches that integrate complementary algorithms/features to ameliorate the estimations of model accuracies. The explicit suitability of a model structure is dictated by its similar features and properties to mimic native or native-like proteins [3], [4], [15]. Considering this, we have developed an integrated approach for determining the quality of decoys to identify the best model structure. The proposed method, ProFitFun-Meta, integrates the features from its antecedent, ProFitFun, with several additional features to facilitate an exceedingly competent and precise estimation of quality of predicted model structures. The potential of ProFitFun-Meta is benchmarked against the recent methods for the estimation of protein structure quality to scrutinize its substantial advancements when tested on the comprehensive protein structure datasets.

## 2 Material and methods

The ProFitFun-Meta was developed by using structural features from the non-redundant datasets of experimentally solved protein structures and predicted model structures of diverse quality. In general, the structures under 3 Å resolution (equivalent to GDT-TS ∼ 0.8 for predicted model structures) deliver the structural information of side chain orientations of amino acid residues in the protein structures and can be regarded as excellent in terms of quality of structures. Likewise, the structure around 5 Å resolution (equivalent to GDT-TS ∼ 0.5 for predicted model structures) can provide the structural information of the overall fold and secondary structural arrangement in the protein tertiary structure and can be considered as good (model) structures. The structural information furnished by the structures beyond 6 Å (equivalent to GDT-TS < 0.3 for predicted model structures) is very limited and thus can be considered as poor in terms of quality of structures. Keeping the extent of structural information available at different resolutions and the rationale of maintaining the structural diversity in terms of quality, a non-redundant initial dataset was compiled from excellent quality experimentally solved protein structures, and variable quality (excellent, good and poor) predicted model structures, as described below.

### 2.1 Dataset compilation

A non-redundant dataset of experimentally solved high resolution (up to 3 Å) globular protein structures was compiled at 90 % sequence identity from Protein Sequence Culling Server (PISCES), resulting in 8041 protein structures [24]. The other filters applied for compiling this dataset were sequence length ranging from 50 to 1000 residues, presence of missing or non-standard residues, and presence of membrane protein. Additionally, a dataset of 87,806 predicted model structures of diverse quality was also compiled by implementing the above mentioned filters from the publicly available decoys of CASP protein targets (CASP9 – CASP14, https://predictioncenter.org). For the CASP experiment, multiple model structures (referred as ‘decoys’) that are predicted through different protein structure prediction methods are available for individual protein sequence (referred as ‘protein target’). The predicted model structures (n = 87,806) in the compiled initial dataset represented 392 protein targets with an average of 205 (±18) model structures for an individual protein. A summary of the distribution of the GTD-TS (global distance test template score) for the predicted model structures (n = 87,807) is shown in supplementary Fig. S1.

### 2.2 Training and Test datasets

The compiled dataset of experimental protein structures and predicted model structures was divided into training and test datasets. Out of 8041 high resolution experimental protein structures, 6030 protein structures were included in the Training Dataset and 2011 protein structures were considered as the Test Dataset. Among the predicted model structures (n = 87,806) of 392 protein targets from CASP9 to CASP14, the model structures (n = 63,213) corresponding to 294 protein targets were included in the Training Dataset and model structures (n = 24,593) corresponding to 98 protein targets were allocated to the Test Dataset. Altogether, the Training Dataset comprised of 69,243 protein structures (experimental and model structures) and the Test Dataset comprised of 26,604 protein structures (experimental and model structures).

### 2.3 Features compilation

For the development of ProFitFun-Meta, the sequence and structure based features from three different quality assessment methods were integrated, namely ProFitFun [20], MolProbity [25], and Procheck [26]. A total of 19 features were adopted from these quality assessment methods, 11 features from ProFitFun, 5 features from MolProbity, and 3 features from Procheck. The features adopted from ProFitFun included three features attributing to the backbone dihedral preferences of amino acid residues and their secondary structural elements, six features referring to the surface accessibility preferences of amino acid residues and their secondary structural elements, one feature representing the sequence to the secondary structure fitness score, and one feature accounting for the average score from other 10 features of ProFitFun. The five features reaped from MolProbity included the fraction of rotamer outliers, the fraction of favored rotamers, the percentage of C_α_ geometry outliers, the normalized clash score per 100 residues, and the overall molprobity score. The three features acquired from Procheck included the G-scores for the dihedral angles, the covalent interactions, and the overall G-score. Notably, all the features integrated in ProFitFun-Meta were derived in pure single model mode. The scores for the selected features were calculated for the compiled datasets of experimental and predicted protein structures. The Pearson correlation of individual features is summarized in supplementary Table S1.

### 2.4 Development of machine learning model from compiled features

**Fig. 1:**
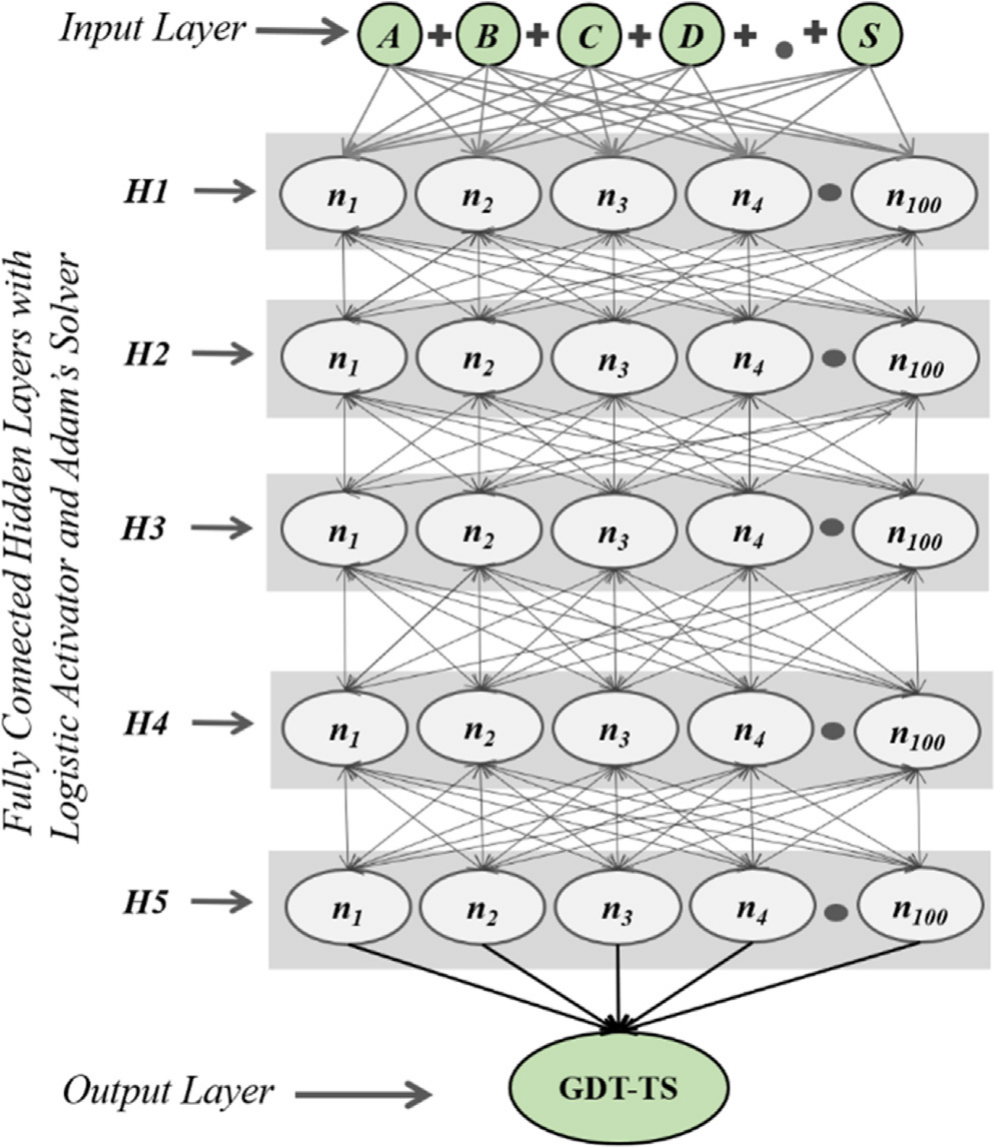
A schematic representation of the multi-layer perceptron with 5 fully connected hidden layers having 100 neurons each with Logistic activator (the logistic sigmoid function) and Adam’s solver (stochastic gradient-based optimizer) function implemented in ProFitFun-Meta.

A neural network model, ProFitFun-Meta, was developed by implementing a replicable training scheme on the Training Dataset (n = 69,243) with a maximum number of 500 iterations through a multi-layer perceptron algorithm with a backpropagation approach having 5 fully connected hidden layers of 100 neurons (Fig. 1). The combinations of different activator and solver functions were used to identify the best parameters. The assessment of the cross validation (10-fold) using different activation and solver functions is outlined in the supplementary Table S2. The best combination of activation function for the hidden layers and solver function for the weight optimization was selected as a function of Mean Square Error (MSE), Mean Absolute Error (MAE) and Pearson’s Correlation Coefficient (r) for the GDT-TS score prediction in the cross validation. Notably, the combination of Logistic activator (the logistic sigmoid function) and Adam’s solver (stochastic gradient-based optimizer) function showed the best evaluation statistics in cross validation for the development of ProFitFun-Meta.

### 2.5 Benchmarking of ProFitFun-Meta

The prediction accuracy of ProFitFun-Meta was evaluated on the Test Dataset (n = 26,604), comprising a completely exclusive dataset of experimentally solved protein structures (n = 2,011) and model structures (n = 24,593). Additionally, the evaluation of ProFitFun-Meta was performed on an external dataset of 40,000 model structures corresponding to 200 non-homologs proteins [27]. The performance of ProFitFun-Meta was benchmarked against some of the latest methods for protein structure quality assessment [20], [28], [29], [30], [31], [32]. A brief description of these methods is provided in supplementary Table S3. The methodological details, their respective publications can be referred as cited in the benchmarking tables (Table 1, Table 2) in results section below.

**Table 1:** A summary of target-wise ProFitFun-Meta performance evaluation and benchmarking on Test Dataset (n = 24,593) using the Pearson’s (r) and Spearman’s (ρ) correlation coefficients, average absolute loss (d), and the average GDT-TS loss (g) between predicted and corresponding observed values of GDT-TS.

Method	Pearson’s CC (r)	Spearman’s CC (ρ)	Absolute loss (d)	GDT-TS loss (g)
ProFitFun-Meta	0.747 ± 0.162	0.698 ± 0.133	0.128 ± 0.103	0.132 ± 0.108
ProFitFun [20]	0.701 ± 0.174	0.653 ± 0.136	0.139 ± 0.118	0.144 ± 0.119
ProQ3D [28]	0.716 ± 0.178	0.661 ± 0.181	0.155 ± 0.098	0.172 ± 0.107
VoroMQA [29]	0.637 ± 0.151	0.619 ± 0.191	0.188 ± 0.145	0.220 ± 0.143
QProb [31]	0.698 ± 0.185	0.652 ± 0.168	0.163 ± 0.102	0.175 ± 0.098
DeepQA [32]	0.684 ± 0.161	0.633 ± 0.177	0.182 ± 0.166	0.194 ± 0.123

**Table 2:** A summary of target-wise ProFitFun-Meta performance evaluation and benchmarking on an External Dataset (n = 40,000) using the Pearson’s (r) and Spearman’s (ρ) correlation coefficients, average absolute loss (d), and the average GDT-TS loss (g).

Method	Pearson’s CC (r)	Spearman’s CC (ρ)	Absolute Loss (d)	GDT-TS Loss (g)
ProFitFun-Meta	0.868 ± 0.104	0.866 ± 0.098	0.075 ± 0.022	0.106 ± 0.041
ProFitFun [20]	0.833 ± 0.110	0.839 ± 0.113	0.072 ± 0.024	0.107 ± 0.044
ProQ3D [28]	0.832 ± 0.151	0.819 ± 0.144	0.108 ± 0.068	0.112 ± 0.078
VoroMQA [29]	0.811 ± 0.119	0.820 ± 0.147	0.192 ± 0.069	0.211 ± 0.071
QProb [31]	0.782 ± 0.125	0.799 ± 0.151	0.110 ± 0.059	0.168 ± 0.076
DeepQA [32]	0.758 ± 0.095	0.787 ± 0.096	0.114 ± 0.102	0.131 ± 0.067

### 2.6 Parameters of performance evaluation

For the performance evaluation and benchmarking of ProFitFun-Meta, the evaluation metrics were adopted from the CASP experiments [15]. The accuracy of the participating methods in CASP experiments is mainly evaluated on the basis of Spearman’s (ρ) and Pearson’s (r) correlation coefficients, GDT-TS loss (g), and absolute loss (d). The absolute loss (d) reflects the measure of error by which the prediction of GDT-TS by the method deviates from the actual observed GDT-TS for the top ranked model by the method (*d = |GDT_Pred_ - GDT_Obs_|*). The GDT-TS loss (g) reflects the measure of error by which the method misses the best model available in the pool of decoys for a protein target (*g = |GDT_TR_ – GDT_BM_|*). The *GDT_TR_* is the predicted GDT-TS of the top ranked model by the used method and *GDT_BM_* is the actual GDT-TS score of the best model available in the evaluated model structure of a protein. Notably, this metric can be implemented in case of rank ordering different models (also called decoys) for the same protein.

## 3 Results and discussion

The evaluation of the ProFitFun-Meta for the prediction of GDT-TS was performed on the basis of the statistics on the Test Dataset (n = 26,604) and an External Dataset (40,000 model structures of 200 non-homolog proteins).

### 3.1 ProFitFun-Meta cross-validation, evaluation and benchmarking on test dataset

The ProFitFun-Meta was initially evaluated on the basis of its performance in the cross validation (10-fold) and the prediction statistics on the Test Dataset. In the cross validation, the ProFitFun-Meta performed exceptionally well with a Pearson’s correlation coefficient (r) of 0.88 (RMSE = 0.103). A similar result could be replicated when the ProFitFun-Meta was evaluated on the Test Dataset and a slight decline in the performance was observed (r = 0.76, RMSE = 0.164). Commonly, the machine learning based trained models tend to underperform when evaluated on a never seen dataset as compared to their performance in cross validation. In the case of ProFitFun-Meta, the Test Dataset was completely exclusive at protein sequence and structural levels as homologous sequences/structures were deliberately eliminated. The performance of ProFitFun-Meta was benchmarked against different state-of-the-art methods on the basis of Pearson’s (r) and Spearman’s (ρ) correlation coefficients, absolute loss (d) and GDT-TS loss (g) metrics, where the high values Pearson’s (r) and Spearman’s (ρ) correlation coefficients indicate a better efficiency of the method to identify and quantify the quality of protein model structures. Likewise, the lower values of absolute loss (d) and GDT-TS loss (g) metrics reflect better performance.

In the case of experimental structures (n = 2,011) in the Test Dataset, only absolute loss (d) from the predicted and observed GDT-TS could be calculated (0.09 ± 0.062). The Pearson’s correlation coefficient could not be calculated as the GDT-TS for all the experimental structures was the same (i.e. 1.0). While the Spearman’s correlation coefficient (ρ), GDT-TS loss (g) and Z-scores could not be calculated due to unavailability of multiple models for the same protein. The performance of ProFitFun-Meta and its benchmarking with other state-of-the-art methods on the Test Dataset (excluding experimental structures) is summarized in Table 1. A brief description of these methods and their mode of the protein structure quality assessment is provided in supplementary Table S3.

The ProFitFun-Meta demonstrated an improved performance in terms of Pearson’s correlation coefficient (r = 0.75), Spearman’s correlation coefficient (ρ = 0.70), absolute loss (d = 0.128), and GDT-TS loss (g = 0.132) over all other methods used in the benchmarking. The ProFitFun-Meta performed significantly better over its antecedent ProFitFun (r = 0.70, ρ = 0.65, d = 0.139, and g = 0.144), that indicated the complementary effect of additional features in ProFitFun-Meta in achieving better accuracy for predicted GDT-TS scores of protein model structures. The significance of ProFitFun-Meta performance was statistically analyzed by a two-sample Kolmogorov-Smirnov test. The statistical analysis supported the significant difference (p-value ≤ 0.001) in performance of ProFitFun-Meta over other methods used in the benchmarking, as summarized in supplementary Table S4.

### 3.2 ProFitFun-Meta evaluation and benchmarking on external dataset

A target-wise evaluation of ProFitFun-Meta was performed on a structurally diverse External Dataset of 40,000 model structures of 200 non-homologous proteins, generated through 3DRobot [27]. The ProFitFun-Meta showed an improved performance over its antecedent, ProFitFun, as well as all the other methods used for the benchmarking. It was observed that the overall performances in terms evaluation statistics for all the methods improved as compared to the corresponding values on the Test Dataset. The results of ProFitFun-Meta evaluation and benchmarking on the External Dataset are summarized in Table 2.

The ProFitFun-Meta method demonstrated an improved overall performance as compared to all the other quality assessment methods used for the benchmarking. The improvement in the performance of ProFitFun-Meta over its predecessor, ProFitFun, could be attributed to the addition of new features.

### 3.3 ProFitFun-Meta evaluation and benchmarking on CASP14 dataset

The benchmarking of the ProFitFun-Meta was further extended with the leading state-of-the-art methods in CASP14, as evaluated by the CASP assessors in the single models QA category.

(https://predictioncenter.org/casp14/doc/presentations/2020_12_03_EMA_Assessment_Seok.pdf). The details of these methods can be accessed from the abstract book of CASP14 proceedings available at https://predictioncenter.org/casp14/doc/CASP14_Abstracts.pdf. Out of total 34 protein targets for which the native structures are available in the public domain, the model structures (n = 3538) of 25 protein targets were part of the Training Dataset, the model structures (n = 1200) for another 8 protein targets were part of Testing Dataset (evaluation statistics were unavailable for one protein target). Therefore, we only extracted the performance metrics of ProFitFun-Meta for these 8 proteins from the Testing Dataset, and that of the leading state-of-the-art methods for the same set of proteins from the CASP14 experiment, https://predictioncenter.org/casp14/results.cgi. The performance benchmarking of ProFitFun-Meta with the leading QA methods of CASP14 experiments is summarized in Table 3.

**Table 3:** A summary of the ProFitFun-Meta evaluation and benchmarking with the leading state-of-the-art methods in quality assessment category of CASP14 experiment.

Method	Pearson’s CC (r)	Spearman’s CC (ρ)	Absolute Loss (d)	GDT-TS Loss (g)
ProFitFun-Meta	0.63 ± 0.15	0.69 ± 0.18	0.107 ± 0.093	0.157 ± 0.129
BAKER-ROSETTA	0.41 ± 0.49	0.43 ± 0.48	0.140 ± 0.068	0.212 ± 0.163
tFold-IDT	0.40 ± 0.30	0.35 ± 0.34	0.279 ± 0.180	0.194 ± 0.156
VoroCNN-GDT	0.42 ± 0.27	0.42 ± 0.30	0.138 ± 0.059	0.196 ± 0.151
ModFold8	0.40 ± 0.43	0.40 ± 0.48	0.110 ± 0.071	0.181 ± 0.155
ProQ3D	0.33 ± 0.36	0.36 ± 0.41	0.128 ± 0.055	0.193 ± 0.177

The ProFitFun-Meta method showed the best performance (r = 0.63, ρ = 0.69, d = 0.107, and g = 0.157), followed by BAKER-ROSETTA (r = 0.41, ρ = 0.43, d = 0.140, and g = 0.212), tFold-IDT (r = 0.40, ρ = 0.35, d = 0.279, and g = 0.194), and VoroCNN-GDT (r = 0.42, ρ = 0.42, d = 0.138, and g = 0.196). Notably, the performance of leading methods in CASP14 quality assessment category was falling within a narrow ranges of Pearson’s correlation coefficient (r = 0.33 to 0.42) and Spearman’s correlation coefficient (ρ = 0.35 to 0.43). It was observed that the ProFitFun-Meta method performed considerably better in terms of Pearson’s correlation coefficient (r), Spearman’s correlation coefficient (ρ), absolute loss (d), and GDT-TS loss (g) as compared to the other methods on the CASP14 dataset.

### 3.4 Computational requirement, implementation, and application

ProFitFun-Meta requires 8–10 min to evaluate 100 model structures of a protein with 300 residues on a single processor. ProFitFun-Meta can be implemented in both the command line mode (for Linux OS based users) or the graphic user interface (for Windows OS based users). Further, ProFitFun-Meta is freely available at http://github.com/KYZ-LSB/ProFitFun-Meta for standalone installations. A detailed and easy to follow user manual for installation requirements and procedure is included therein. MolProbity and Procheck are not distributed with the standalone version of ProFitFun-Meta due to license related restrictions and can be obtained from the respective developers. However, details related to their download and installation are provided for the convenience of the users.

## 4 Conclusion

The recent innovative methodological developments in the field of protein structure prediction are mainly by the virtue of various machine learning based approaches. The field of computational protein structural modeling and protein design have witnessed the implementation of different flavors of machine learning approaches to deliver a remarkable success. The revolutionary success of some advanced deep-learning based state-of-the-art methods has demonstrated a promising potential of computationally generated protein structural information in substantiating its impact in computer aided drug discovery, computational protein design and protein functional characterization. The success of protein structural modelling and computational protein design depends upon the reliable protein structure quality assessment. However, the accuracy estimation of the predicted model structure has not yet been consummated adequately. The proposed method here, ProFitFun-Meta, is intended to serve the protein structure biologists in optimally employing and comprehending the predicted protein structural information. We integrated the structural information of experimentally characterized proteins, in terms of backbone dihedral angles and surface accessibility preferences of residues, spatial properties of protein structures for quality estimation (side chain and backbone clashes, rotamer orientations, and Cα geometry), and potential of model structures to mimic the experimentally solved protein structures by accounting for the deviations of covalent interactions, dihedral angles and overall interactions. These parameters were unified together through a neural network based machine learning model, ProFitFun-Meta, to deliver a highly precise quality evaluation of the protein model structures. The accuracy of ProFitFun-Meta is validated on an extensive dataset of ∼ 65,000 model structures of non-homologous proteins.

A better knowledge of structural and functional features of proteins is pivotal to various therapeutic developments in identifying novel drug targets. ProFitFun-Meta can furnish a reliable quantification of the structural quality of the predicted model structures that are generated through different protein structure prediction methodologies. For instance, the reliable scoring of model structures can expedite an optimal utilization of structural models for computer aided drug discovery regimes, ligand binding studies, structure to function characterization, and various protein–protein interaction and mutagenesis studies. The accuracy of ProFitFun-Meta can be easily harnessed by incorporating it into the computational pipelines for protein modeling and protein design. The minimal additional dependencies, high time and computational efficiency, and the automated standalone source codes optimized for portability to various Linux and Windows OS provide an additional edge to ProFitFun-Meta for its potential applications in various regimes of computational protein design, computational protein folding and function annotation, and computer aided drug designing for the scientists even without computational biology and bioinformatics expertise.

## Declaration of Competing Interest

The authors declare that they have no known competing financial interests or personal relationships that could have appeared to influence the work reported in this paper.
