# Supplementary material for: An integrated protein structure fitness scoring approach for identifying native-like model structures
Source: Comput Struct Biotechnol J. 2022 Nov 17;20:6467–72. doi: 10.1016/j.csbj.2022.11.032 (PMC9708444; doi:10.1016/j.csbj.2022.11.032)
Supplement: Supplementary data 1 [file mmc1.docx]

**Supplementary Information**

**An Integrated Protein Structure Fitness Scoring Approach for Identifying Native-Like Model Structures**

Rahul Kaushik^1, #^ and Kam Y. J. Zhang^1, *^

^1^Laboratory for Structural Bioinformatics, Center for Biosystems Dynamics Research, RIKEN, 1-7-22 Suehiro, Yokohama, Kanagawa 230-0045, Japan

^#^Present Address: Biotechnology Research Center, Technology Innovation Institute, P.O. Box 9639, Masdar City, Abu Dhabi, UAE

^*^Corresponding author (kamzhang@riken.jp)

**Short Title: Integrated Protein Structure Fitness Function**

**Supplementary Tables**

**Table S1.** A summary of Pearson correlation coefficients of different sequence and structural features with GDT-TS for the protein structures accounted in the Training Dataset.

| S.N. | Sequence and Structural Features | r (GDT-TS) |
| --- | --- | --- |
| 1 | Sequence and Sec Str. Based Competency Scores | 0.30 |
| 2 | Backbone Dihedral Preferences – Amino Acid (AA) Triplets | 0.25 |
| 3 | Backbone Dihedral Preferences – Sec. Str. (SS) Triplets | 0.38 |
| 4 | Backbone Dihedral Preferences – AA and SS Triplets | 0.51 |
| 5 | Main and Side Chain SA Preferences – Amino Acid (AA) Triplets | 0.44 |
| 6 | Main and Side Chain SA Preferences – Sec. Str. (SS) Triplets | 0.58 |
| 7 | Main and Side Chain SA Preferences – AA and SS Triplets | 0.64 |
| 8 | Polar and Non-Polar Atoms SA Preferences – Amino Acid Triplets | 0.57 |
| 9 | Polar and Non-Polar Atom SA Preferences – Sec. Str. Triplets | 0.56 |
| 10 | Polar and Non-Polar Atom SA Preferences – AA and SS Triplets | 0.62 |
| 11 | Overall quality score (QS_P_) | 0.63 |
| 12 | Fraction of rotamers outliers | -0.27 |
| 13 | Fraction of favored rotamers | 0.22 |
| 14 | Percentage of Cα geometry outliers | -0.35 |
| 15 | Normalized clash score | -0.46 |
| 16 | Normalized MolProbity score | 0.37 |
| 17 | G-score for dihedral angles | 0.29 |
| 18 | G-score for covalent interactions | 0.27 |
| 19 | Overall G-score | 0.32 |

**Table S2.** A summary of Root Mean Square Error (RMSE), Mean Absolute Error (MAE) and Pearson’s Correlation Coefficient (r) in 10-fold cross validation for the prediction of GDT-TS by implementing different combinations of activator (in column 1) and solver (in row 1) functions. The three comma separated values in each cell corresponds to RMSE, MAE and r for respective combination.

|  | Adam | SGD^1^ | L-BFGS-B^2^ |
| --- | --- | --- | --- |
| ReLu | 0.103, 0.076, 0.86 | 0.167, 0.132, 0.77 | 0.134, 0.101, 0.85 |
| Logistic | *0.103, 0.072, 0.88* | 0.192, 0.171, 0.64 | 0.174, 0.137, 0.75 |
| tanh | 0.119, 0.087, 0.83 | 0.171, 0.135, 0.76 | 0.149, 0.115, 0.82 |
| Identity | 0.192, 0.151, 0.69 | 0.189, 0.152, 0.69 | 0.188, 0.151, 0. 70 |
| ^1^Stochastic Gradient Descent (SGD) is an approach to fitt linear classifiers and regressors under convex loss functions such as Support Vector Machines and Logistic Regression.  ^2^It is an optimization algorithm to approximate the Broyden–Fletcher–Goldfarb–Shanno algorithm (BFGS) by implementing inverse Hessian matrix to search through variable space. The L-BFGS-B extends BFGS to handle simple box constraints with limited memory. | | | |

**Table S3.** A brief description and the mode of quality assessment of the methods used for benchmarking the performance of ProFitFun-Meta.

| Method | Quality Assessment | Overview of Method |
| --- | --- | --- |
| ProFitFun | Global | ProFitFun implements the backbone dihedral and residue surface accessibility preferences through neural networks to deliver a reliable protein structure quality assessment. |
| ProQ3D | Local and Global | ProQ3D is the successor of ProQ2 and ProQ3 and it implements deep neural networks to perform local and global quality assessment of protein structures. |
| VoroMQA | Local and Global | VoroMQA implements statistical potentials with Voronoi tessellation of atomic balls to estimate the protein structure quality in terms of atomic, residue and global scores. |
| QProb | Global | QProb computes the absolute error for the protein structural features against the true quality scores to estimate the probability density distribution for quality assessment. |
| DeepQA | Global | DeepQA implements deep belief networks by utilizing physico-chemical and structural information of proteins to deliver global protein structure quality assessment. |

**Table S4.** A summary of two sample Kolmogorov-Smirnov test (significance level (α) = 0.001) for the comparison of Pearson’s correlation coefficient (r), Spearman’s correlation coefficient (ρ), absolute loss (d), and GDT-TS loss (g) of ProFitFun-Meta and other methods.

| Methods | Pearson’s CC (r) | Spearman’s CC (ρ) | Absolute Loss (d) | GDT-TS Loss (g) | |
| --- | --- | --- | --- | --- | --- |
| ProFitFun | 0.243, < 0.001 | 0.272, < 0.001 | 0.298, < 0.0001 | 0.325, < 0.0001 | |
| VoroMQA | 0.268, < 0.0001 | 0.383, < 0.0001 | 0.621, < 0.0001 | 0.613, < 0.0001 | |
| DeepQA | 0.477, < 0.0001 | 0.491, < 0.0001 | 0.693, < 0.0001 | 0.618, < 0.0001 | |
| QProb | 0.393, < 0.0001 | 0.288, < 0.0001 | 0.703, < 0.0001 | 0.684, < 0.0001 | |
| ProQ3D | 0.227, < 0.001 | 0.268, < 0.001 | 0.312, < 0.0001 | 0.344, < 0.0001 | |
| Interpretation: No difference among the performance of ProFitFun and other methods was considered as null hypothesis. The maximum difference (D) between the two samples (evaluation metrics of ProFitFun-Meta and other method) and p-values are reported. If the p-value is greater than the significance value (0.001), the null hypothesis may be accepted, i.e. there is no significant difference among the performance of two methods for the corresponding evaluation metric. | | | | |  |

**Supplementary Figures**

**
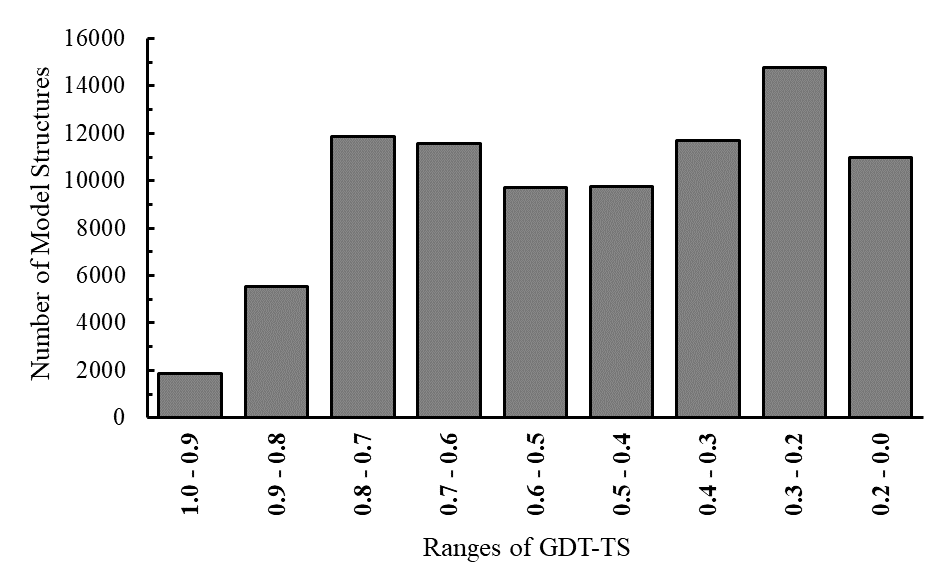
Figure S1.** Distribution of global distance test based template scores (GDT-TS) for the compiled dataset of 87,806 decoy structures of 392 proteins adopted from different CASP experiments.
